# Thermal stratification effect on gravity driven transport of hybrid CNTs down a stretched surface through porous medium

**DOI:** 10.1016/j.heliyon.2023.e15699

**Published:** 2023-04-22

**Authors:** Faisal Z. Duraihem, E.N. Maraj, Noreen Sher Akbar, R. Mehmood

**Affiliations:** a Department of Mathematics, College of Science, King Saud University, P.O. Box 2455, Riyadh, 11451, Saudi Arabia; b Department of Mathematics, National Skills University, Islamabad, 44000, Pakistan; c DBS&H, CEME, National University of Sciences and Technology, Islamabad, Pakistan; d Department of Mathematics, HITEC University Taxila, Pakistan

**Keywords:** Hybrid carbon nanotubes, Thermal sensitive surface, Porous medium, Gravity-driven flow

## Abstract

The purpose of the current article is to explore the impact of thermal stratification and medium porosity on gravity-coerced transport of hybrid carbon nanotubes down an upright extending sheet inspired by a constant applied magnetic field along with heat transfer investigation in existence of thermal radiation, viscous dispersal, and joule heating effect. Rectangular coordinates are chosen for the mathematical interpretation of the governing flow problem. Homothetic analysis is employed for the sake of simplification process. The reduced system of coupled nonlinear differential equations is dealt numerically by dint of computational software MATLAB inbuilt routine function Bvp4c. The numerical investigation is carried out for the distinct scenarios namely, (i) Presence of favorable buoyancy force, (ii) Case of purely forced convection and (iii) Presence of opposing buoyancy force. Significant Findings: The key findings include that the presence of hybrid carbon nanotubes and medium porosity contributes significantly to upsurging surface shear stress magnitude whereas, external magnetic field and velocity slip effects in an altered manner. The present study may be a benchmark in study of fueling process in space vehicles and space technology.

## 1 Introduction

Numerous Industrial and manufacturing processes involve liquid thin film flow along solid surfaces. Typical examples of such gravity-driven vertical flows are heat exchangers, turbine blades, refrigeration processes, evaporators, condensers, and trickling filters [1]. Due to its wide range of applications, thin-film flows have been and still are a subject of keen interest for the past few decades. Andersson and Irgens [2] examined the thin film transport of power-law fluids. Pop et al. [3] discussed gravity-driven film flow along an upright wall under the consideration of surface mass transfer and later inspected the coupled film flow towards a heated standing solid surface [4]. Shang et al. [5] extended the work of Andersson and Irgens by explored the hydrodynamics effects and provide accurate numerical solutions for the power-law index n. Variable physical properties of thin-film flow have been investigated by Andersson et al. [6]. Their study showed that momentum and thermal boundary layer thickness shrinks with enhancing wall temperature. Luo and Pozrikidis [7] considered the inclined wall case with three-dimensional corrugations to inspect gravity-driven film flow. The obtained results revealed the surface velocity field and surfactant distribution and consideration of three-dimensional wall geometry led to reducing the surface deformation. Meza et al. [8] presented the thermodynamic analysis of the thin liquid film along with an inclined heat plate. They considered the hydromagnetic and viscous dissipation effects in their study and found out that entropy generation number increases with Hartman number and Brinkman number. Kartini et al. [9] deliberated on micropolar fluid film flow and presented the Falkner-Skan solution. The computed results revealed that the coefficient of skin friction is positively related to the material parameters.

Insertion of ultrafine nanoparticles within traditional base fluids for instance ethylene glycol, water, kerosene oil, Engine Oil, and Propylene-Glycol significantly improves the thermal efficiency of such working fluids. In numerous industrial and technological processes such as power generation, glass fiber, automobiles, electronics, heat exchangers, and polymer extrusion processes involve the usage of nanofluids as an efficient cooling agent [[10], [11], [12], [13], [14]]. In the past few decades, scientists are keenly interested in exploring the impact of inserting carbon nanotubes within traditional base fluids having weak thermal capabilities. Carbon nanotubes (CNTs) are cylindrically shaped molecules consisting of single-layer or multi-layer graphene atoms rolled-up sheets. Their length can vary from micrometers to millimeters. Several promising properties of CNTs such as increased tensile strength, improved heat deflection temperature, better electrical conductivity, and unique chemical properties make them an optimal choice for improving the heat transfer capabilities of working fluids [15]. CNTs find promising applications in solar cells, ion batteries, field emission displays, nanotube sensors, and transistors. Wang et al. [16] presented the idea of thermal conductivity enhancement with carbon nanotubes. Choi et al. [17] inserted the multi-walled carbon nanotubes in the base fluid to improve nanofluid thermal conductivity. The idea of homogeneous CNTs utilization in electrical applications was coined by Ramasubramaniam [18]. Later on, Wang et al. [19] incorporated carbon nanotubes to explore nanofluid's pressure drop and heat transfer aspect.

Magnetohydrodynamics (MHD) has been a subject of keen interest due to its existence in several phenomena such as liquid metal, plasma confinement, electromagnetic casting, and many more. Several studies have been conducted on MHD convection in nanofluids [[20], [21], [22], [23]]. During the past decade or so, a lot more focus has been on the role of Carbon nanotubes in MHD natural convection base fluids. Ellahi et al. [24] studied the MHD natural convection in saltwater using single and multi-wall CNTs. They concluded that by enhancing Rayleigh number and volumetric fraction, heat transfer rate is significantly improved. Malvandi et al. [25] developed the MHD flow of water base fluid in presence of multi-walled nanotubes within a microchannel and observed that Carbon nanotubes have a positive influence on the heat transfer capabilities along the microchannel walls. Maraj et al. [26] explored the influence of MHD on carbon nanotubes suspensions having erratic thermophysical attributes. They found out that Nusselt number is significantly enhanced with volume fraction, radiation parameter, and viscosity parameter. Later, they conducted another similar kind of study to discover the contribution of engendered magnetic field on Propylene Glycol-based fluid in the presence of CNTs [27].

The above-cited literature review reveals that although CNTs based fluids are seeking a lot of attention from researchers for the last decade or so. Yet it is quite obvious that the gravity-driven flow of MHD hybrid flow of CNTs based fluid (Containing both single and multiwall carbon nanotubes) has not been given much attention. For physically realistic analysis, velocity slip and convective heating conditions at the wall are taken into consideration. Moreover, Fluid suction/Injection phenomena have been included in the computational study as it not only influences the flow and thermal characteristics but due to its occurrence by leakage within the wall which can occur naturally as well or it can be intentional for the purpose of controlling boundary-layer characteristics from the wall. The flow governing problem is modeled and transformed by means of scaling analysis. The transformed system is tackled numerically. Physically quantities are portrayed against sundry parameters and are supported with physical discussion. The concluding remarks are presented at the end section. Such flows are of immense importance in space technology and design of space vehicles. The present investigation may be beneficial in academics research and is a benchmark for gravity driven flow problems encountered in environmental and chemical engineering.

## 2 Mathematical description

**Fig. 1:**
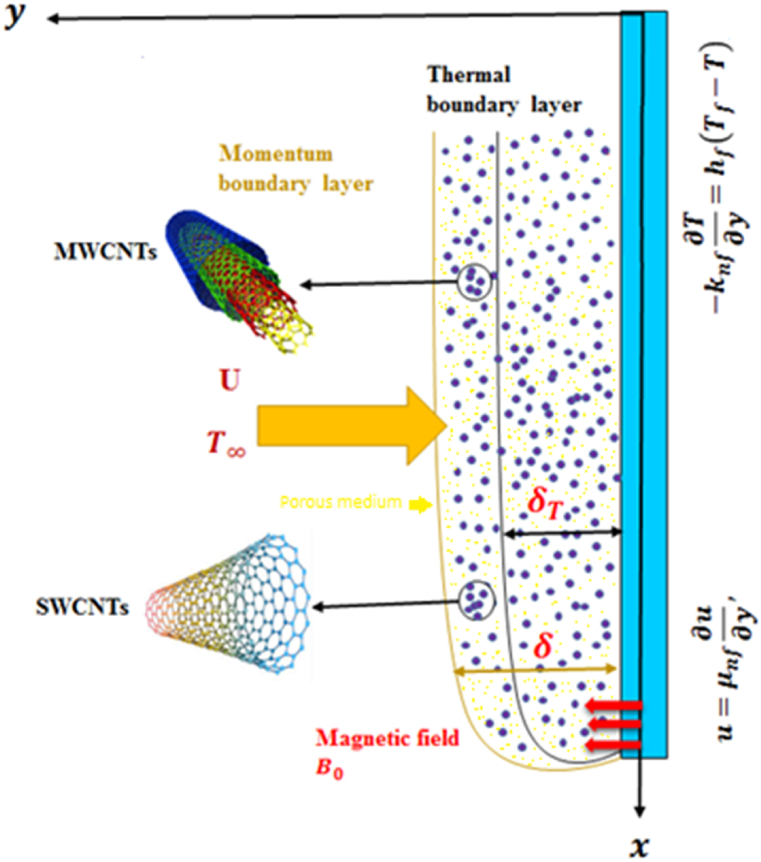
Schematic diagram.

Consider the two-dimensional hybrid Carbon nanotubes (CNTs) gravity coerced thin film transport through a porous medium passed over a vertically stretching surface influenced by external magnetic field of constant magnitude $B_{0}$ applied normal to the surface and mixed convection. Here single wall (SW) and multi-wall (MW) carbon nanotubes are considered to be suspended in water $H_{2}O$ considered to be the base fluid. The partial slip effect at the surface influenced the transport of hybrid CNTs embedded in $H_{2}O$ and the free stream velocity far away from the sheet is taken to be $U\left(x\right)=\sqrt{2gx}$. Moreover, heat transfer analysis is performed by taking into account the thermal radiation, viscous dispersal and joule heating effect along with convective boundary and a peripheral temperature is taken to be $T_{\infty }=T_{0}<T_{f}$, here subscript $T_{f}$ stands for fluid temperature. The mathematical derivation of the physical flow problem is carried out in rectangular coordinate system. The x− axis is assumed to be in earth gravity $\left(\vec{g}\right)$ direction along the upright stretching sheet and y− axis is taken normal to the surface. Fig. 1 illustrates the schematic diagram of flow problem.

In the current investigation, hybrid CNTs nanofluid is taken to be the mixture of SWCNTs into MWCNTs/Water. Firstly, the nm size MWCNTs of volumetric fraction $\left(\varphi_{1}\right)$ are added in water to form a MWCNTs/water nanofluid then SWCNTs of volumetric fraction $\left(\varphi_{2}\right)$ are inserted in the nanofluid to acquire a homogeneous uniform amalgam of hybrid nanofluid SWCNTs−MWCNTs/water. The pressure gradient term in view of Oberbeck –Boussinesq approximation is defined as follows.

(1) $$\frac{dp}{dx}=\rho_{hnf}\;U\frac{dU}{dx}\;and\;U\left(x\right)=\sqrt{2gx}$$

By invoking boundary layer approximation, the governing system of equations takes the following form (see Refs. [3,19]):

(2) $$\frac{\partial u}{\partial x}+\frac{\partial v}{\partial y}=0\text{,}$$

(3) $$\rho_{hnf}\left[u\frac{\partial u}{\partial x}+v\frac{\partial u}{\partial y}\right]=\mu_{hnf}\;\frac{\partial^{2}u}{\partial y^{2}}-\mu_{hnf}\frac{1}{k_{1}}u+\beta_{hnf}g\left(T-T_{\infty }\right)-{\tilde{\sigma }}_{hnf}B_{0}^{2}u\text{,}$$

(4) $$u\frac{\partial T}{\partial x}+v\frac{\partial T}{\partial y}=\alpha_{hnf}\frac{\partial^{2}T}{\partial y^{2}}+\frac{1}{{\left(\rho c_{p}\right)}_{hnf}}\left[-\frac{16}{3}\frac{\sigma^{*}T_{\infty }^{3}}{k^{*}}\right]\frac{\partial^{2}\;T}{\partial y^{2}}+\frac{\mu_{hnf}}{{\left(\rho c_{p}\right)}_{hnf}}\;{\left(\frac{\partial u}{\partial y}\right)}^{2}-\frac{{\tilde{\sigma }}_{hnf}B_{0}^{2}}{{\left(\rho c_{p}\right)}_{hnf}}u^{2}\text{,}$$

With the associated boundary conditions defined as

$$u=\mu_{hnf}L\frac{\partial u}{\partial y},-k_{hnf}\frac{\partial T}{\partial y}=h_{f}\left(T_{f}-T\right),at\;y=0\text{,}$$

(5) $$u\to U\left(x\right)=\sqrt{2gx},T\to T_{0}\;as\;y\to \infty \text{.}$$

Here Eq [1] is the continuity equation, Eq [2] and Eq [3] are momentum equations in components form the second last and last term in Eq (3) are the mixed convection and Lorentz force contribution, respectively. Eq [4] is the heat equation β is thermal expansion coefficient and T represents the hybrid nanofluid temperature. The subscript hnf stands for hybrid nanofluid and density, dynamic and kinematic viscosities are represented by Greek symbols ρ,μ, and ν, respectively. Corresponding boundary conditions are defined in Eq [5]. Moreover, $\left(\rho c_{p}\right),\alpha \text{,}$ and k are the heat capacitance, thermal diffusivity and conductivity, respectively. The thermophysical attribute's empirical relations (see Ref. [28]) for CNTs are defined as:

$$\frac{\rho_{hnf}}{\rho_{f}}=\left(1-\varphi_{2}\right)\left[\left(1-\varphi_{1}\right)+\varphi_{1}\frac{\rho_{MWCNTs}}{\rho_{f}}\right]+\varphi_{2}\frac{\rho_{SWCNTs}}{\rho_{f}}\text{,}$$

$$\frac{\mu_{hnf}}{\mu_{f}}=\frac{1}{{\left(1-\varphi_{1}\right)}^{2.5}{\left(1-\varphi_{2}\right)}^{2.5}}\text{,}$$

$$\frac{{\left(\rho \beta \right)}_{hnf}}{{\left(\rho \beta \right)}_{f}}=\left(1-\varphi_{2}\right)\left[\left(1-\varphi_{1}\right)+\varphi_{1}\frac{{\left(\rho \beta \right)}_{MWCNTs}}{\left(\rho \beta_{f}\right)}\right]+\varphi_{2}\frac{{\left(\rho \beta \right)}_{SWCNTs}}{\left(\rho \beta_{f}\right)}\text{,}$$

$$\frac{{\left(\rho C_{p}\right)}_{hnf}}{{\left(\rho C_{p}\right)}_{f}}=\left(1-\varphi_{2}\right)\left[\left(1-\varphi_{1}\right)+\varphi_{1}\frac{{\left(\rho C_{p}\right)}_{MWCNTs}}{{\left(\rho C_{p}\right)}_{f}}\right]+\varphi_{2}\frac{{\left(\rho C_{p}\right)}_{SWCNTs}}{{\left(\rho C_{p}\right)}_{f}}\text{,}$$

$$\frac{k_{hnf}}{k_{f}}=\left(\frac{1-\varphi_{1}+2\varphi_{1}\frac{k_{MWCNTs}}{k_{MWCNTs}-k_{f}}\ln\left(\frac{k_{MWCNTs}+k_{f}}{2k_{f}}\right)}{1-\varphi_{1}+2\varphi_{1}\frac{k_{f}}{k_{MWCNTs}-k_{f}}\ln\left(\frac{k_{MWCNTs}+k_{f}}{2k_{f}}\right)}\right)$$

$$\times \left(\frac{1-\varphi_{2}+2\varphi_{2}\frac{k_{SWCNTs}}{k_{SWCNTs}-k_{f}}\ln\left(\frac{k_{SWCNTs}+k_{f}}{2k_{f}}\right)}{1-\varphi_{2}+2\varphi_{2}\frac{k_{f}}{k_{SWCNTs}-k_{f}}\ln\left(\frac{k_{SWCNTs}+k_{f}}{2k_{f}}\right)}\right)\text{,}$$

$$\frac{{\tilde{\sigma }}_{hnf}}{{\tilde{\sigma }}_{f}}=\left(1+\frac{3\varphi_{1}\left(\frac{{\tilde{\sigma }}_{CMWNTs}}{{\tilde{\sigma }}_{f}}-1\right)}{\left(\frac{{\tilde{\sigma }}_{MWCNTs}}{{\tilde{\sigma }}_{f}}+2\right)-\varphi_{1}\left(\frac{{\tilde{\sigma }}_{MWCNTs}}{{\tilde{\sigma }}_{f}}-1\right)}\right)$$

(6) $$\times \left(1+\frac{3\varphi_{2}\left(\frac{{\tilde{\sigma }}_{SWCNTs}}{{\tilde{\sigma }}_{f}}-1\right)}{\left(\frac{{\tilde{\sigma }}_{SWCNTs}}{{\tilde{\sigma }}_{f}}+2\right)-\varphi_{2}\left(\frac{{\tilde{\sigma }}_{SWCNTs}}{{\tilde{\sigma }}_{f}}-1\right)}\right)\text{.}$$

In Eq [6], the base fluid and nm size particles are denoted by the subscripts f and s, respectively. According to Ref. [29], we incorporate Rosseland diffusion approximation to express the radiative heat flux as:

(7) $$q_{r}=-\frac{4}{3}\;\frac{\sigma^{*}}{k^{*}}\frac{\partial T^{4}}{\partial y}=-\frac{16}{3}\frac{\sigma^{*}T_{\infty }^{3}}{k^{*}}\frac{\partial T}{\partial y}$$

here $\sigma^{*}=\left(5.67\times 10^{-8}W/m^{2}K^{4}\right)$ is Stefan Boltzmann constant and $k^{*}\left(m^{-1}\right)$ is Rosseland mean absorption coefficient. Making use of Eq. (7), Eq. (4) takes the form:

(8) $$u\frac{\partial T}{\partial x}+v\frac{\partial T}{\partial y}=\alpha_{hnf}\frac{\partial^{2}T}{\partial y^{2}}+\frac{1}{{\left(\rho c_{p}\right)}_{hnf}}\left[-\frac{16}{3}\frac{\sigma^{*}T_{\infty }^{3}}{k^{*}}\right]\frac{\partial^{2}\;T}{\partial y^{2}}+\frac{\mu_{hnf}}{{\left(\rho c_{p}\right)}_{hnf}}\;{\left(\frac{\partial u}{\partial y}\right)}^{2}-\frac{{\tilde{\sigma }}_{hnf}B_{0}^{2}}{{\left(\rho c_{p}\right)}_{hnf}}u^{2}\text{.}$$

Following the relations defined in Refs. [30,31] we introduce the following non-dimensional similarity variables.

(9) $$\psi \left(x,y\right)=\sqrt{\frac{4Uv_{f}x}{3}}f\left(\eta \right),\eta =y\sqrt{\frac{3U}{4v_{f}x}},\theta \left(\eta \right)=\frac{\left(T-T_{0}\right)}{\left(T_{f}-T_{0}\right)}\text{,}$$

By incorporating above expressions conservation of mass holds and Eqs. (3) and (8) reduce to the following system

$$f^{\prime \prime \prime }\left(\eta \right)-\frac{2}{3}\frac{1}{K}f^{\prime }\left(\eta \right)+\frac{\rho_{hnf}}{\rho_{f}}\;\frac{\mu_{f}}{\mu_{hnf}}(f\left(\eta \right)f^{\prime \prime }\left(\eta \right)+\frac{2}{3}\left(1-f^{\prime 2}\left(\eta \right)\right)+\frac{\rho_{f}}{\rho_{hnf}}\frac{{\left(\rho \beta \right)}_{hnf}}{{\left(\rho \beta \right)}_{f}}\sigma \theta$$

(10) $$-\frac{\rho_{f}}{\rho_{hnf}}\frac{{\tilde{\sigma }}_{hnf}}{{\tilde{\sigma }}_{f}}\;M^{2}f^{\prime }\left(\eta \right))=0\text{,}$$

(11) $$\left(\frac{k_{nf}}{k_{f}}+\frac{4}{3}Rd\right)\theta^{\prime \prime }\left(\eta \right)+\frac{\mu_{nf}}{\mu_{f}}EcPrf^{\prime \prime^{2}}\left(\eta \right)-\frac{4}{3}\frac{{\tilde{\sigma }}_{nf}}{{\tilde{\sigma }}_{f}}M^{2}Ec{Prf}^{\prime 2}\left(\eta \right)+\frac{{\left(\rho C_{p}\right)}_{nf}}{{\left(\rho C_{p}\right)}_{f}}f\left(\eta \right)\theta^{\prime }\left(\eta \right)\mathrm{Pr}=0\text{,}$$

with boundary conditions

$$f^{\prime }\left(\eta \right)=\delta \frac{\mu_{nf}}{\mu_{f}}f^{\prime \prime }\left(\eta \right),\theta^{\prime }\left(0\right)=-\gamma \frac{k_{f}}{k_{nf}}\left(1-\theta \left(0\right)\right),\eta =0$$

(12) $$f^{\prime }\left(\eta \right)\to 1,\theta \left(\eta \right)\to 0,as\;\eta \to \infty$$

here buoyancy force because of temperature difference is denoted by σ, $Gr_{x}$ and $Re_{x}$ stands for local Grashof and Reynolds number, respectively. Magnetic parameter $\left(M^{2}\right)$ , thermal radiation parameter (Rd), Prandtl (Pr) and Eckert (Ec) numbers, velocity slip (δ) parameters, heat transfer coefficient (Biot number γ) and medium porosity (K) parameter, respectively are defined as:

$$\sigma =\frac{\beta_{f}}{3}\left(T_{f}-T_{0}\right),Gr_{x}=\left(\frac{g\beta_{f}\left(T_{w}-T_{\infty }\right)}{v_{f}^{2}}\right),Re_{x}=\left(\frac{Ux}{v_{f}}\right),M^{2}=\frac{{\tilde{\sigma }}_{f}B_{0}^{2}U}{\rho_{f}g},Rd=\left(\frac{4\sigma^{*}T_{\infty }^{3}}{k^{*}k_{f}}\right)\text{,}$$

(13) $$\mathrm{Pr}=\left(\frac{{\left(\rho c_{p}\right)}_{f}v_{f}}{k_{f}}\right),Ec=\frac{U^{2}}{\left(T_{f}-T_{0}\right)c_{p}},\delta =\left(L\mu_{f}\sqrt{\frac{3U}{4v_{f}x}}\right),\gamma =\left(\frac{h_{f}}{k_{f}}\sqrt{\frac{4v_{f}x}{3U}}\right),K=\frac{gk_{1}}{\nu_{f}U}\text{,}$$

The surface drag force and heat flux are defined in Eq [14]:

(14) $$\tau_{w}=\mu_{hnf}{\left(\frac{\partial u}{\partial y}\right)}_{y=0}\;q_{w}=-k_{hnf}{\left(\frac{\partial T}{\partial y}\right)}_{y=0}+{\left(q_{r}\right)}_{w}$$

Moreover, the relevant physical quantities of industrial significance, such as, local skin friction coefficient $C_{f_{x}}$ and local Nusselt number $Nu_{x}$, respectively are expressed in Eq [15]:

(15) $$C_{f_{x}}=\frac{\tau_{w}}{\rho_{f}U_{2}},Nu_{x}=\frac{xq_{w}}{k_{f}\left(T_{f}-T_{0}\right)}\text{,}$$

The above expressions in view of Eq. (9) and Eq [13] takes the following form presented in Eq [16]:

(16) $$C_{f}=\sqrt{Re_{x}C_{f_{x}}}=\frac{\sqrt{3}}{2}\;\frac{\mu_{hnf}}{\mu_{f}}f^{\prime \prime }\left(0\right),Nu=\frac{Nu_{x}}{\sqrt{Re_{x}}}=-\frac{\sqrt{3}}{2}\left(\frac{k_{hnf}}{k_{f}}+\frac{4}{3}Rd\right)\theta^{\prime }\left(0\right)\text{,}$$

## 3 Computational procedure

The simplified system comprising of Eqs. (10) and Eq (11) subject to the boundary conditions.

**Fig. 2:**
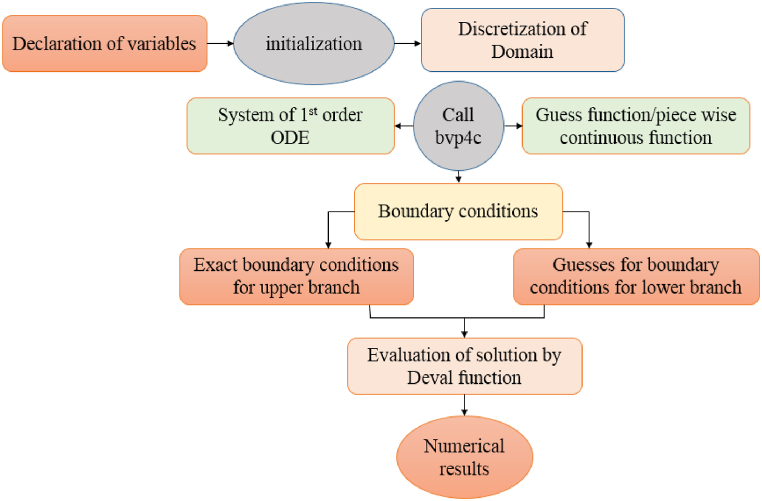
Bvp4c Algorithm.

Eq (12) is dealt numerically by means of computational software MATLAB inbuilt routine function Bvp4c designed on three stage Lobatto IIIa formulation. It is worth mentioning that here class one continuous results achieving accuracy up to six decimals are computed. Fig. 2 is displayed to illustrate the details of procedure followed by Bvp4c algorithm.

Firstly, the system of first order differential equations is formed by reducing higher order differential equations in terms of new variables $w_{i};i=1,2,3,\ldots$ as:

$$w_{1}=f\text{,}$$

$$w_{1}^{\prime }=w_{2}=f^{\prime }\text{,}$$

$$w_{2}^{\prime }=w_{3}=f^{\prime \prime }\text{,}$$

$$w_{3}^{\prime }=\frac{2}{3}\frac{1}{K}w_{2}+\frac{\rho_{f}}{\rho_{hnf}}\;\frac{\mu_{f}}{\mu_{hnf}}\left(\frac{2}{3}\left(w_{2}^{2}-1){-w}_{1}w_{3}\right)-\frac{\mu_{f}}{\mu_{hnf}}\frac{{\left(\rho \beta \right)}_{hnf}}{{\left(\rho \beta \right)}_{f}}\sigma w_{4}+\frac{\mu_{f}}{\mu_{hnf}}M^{2}y_{2}\right)\text{,}$$

$$w_{4}=\theta \text{,}$$

$$w_{4}^{\prime }=w_{5}=\theta^{\prime }$$

(17) $$w_{5}^{\prime }=-\frac{1}{\left(\frac{k_{hnf}}{k_{f}}+\frac{4}{3}Rd\right)}\left(\frac{{\left(\rho C_{p}\right)}_{hnf}}{{\left(\rho C_{p}\right)}_{f}}\mathrm{Pr}\;w_{1}w_{5}+\frac{\mu_{hnf}}{\mu_{f}}EcPrw_{3}^{2}+\frac{4}{3}M^{2}EcPrw_{2}^{2}\right)\text{,}$$

Subject to the boundary conditions are

$$w_{1}\left(0\right)=0,w_{2}\left(0\right)=\delta \frac{\mu_{hnf}}{\mu_{f}}w_{3}\left(0\right),w_{5}\;\left(0\right)=-\gamma \frac{k_{f}}{k_{hnf}}\left(1-w_{4}\left(0\right)\right)$$

(18) $$w_{2}\left(n\right)=1,w_{4}\left(n\right)=0\text{,}$$

here numerical procedure of bvp4c is performed over the interval [0,12] with mesh point 360 for Eq [17]. subject to the boundary conditions presented in Eq [18].

## 4 Graphical results and discussion

This section is devoted to the detailed study of the impact of emerging prominent parameters like Hartmann number, porosity parameter, thermal radiation parameter, slip parameter, Biot number, Eckert number, and volumetric fraction of carbon nanotubes in Hybrid nanofluid on flow velocity and temperature profiles along with physically significant quantities for instance surface drag force and heat transfer rate through graphical illustrations and tabulated results, respectively. The graphs are plotted for three distinct scenarios, that is, (i) when σ>0 (physically this means presence of favorable buoyancy force), (ii) when σ=0 (physically this is case of purely forced convection) and (iii) when σ<0 (physically it represents the presence of opposing buoyancy force. In the graphs displayed below, solid lines indicate the results when σ>0, dotted lines are exercised to display the results for purely free convection and dashed lines represent the results when σ<0, respectively.

### 4.1 Velocity distribution

**Fig. 3:**
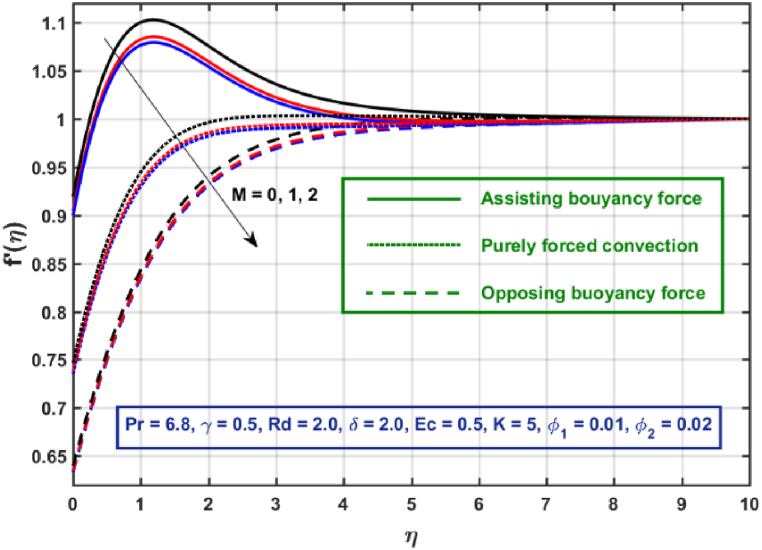
Reprsent vekocity profile for different values of hartmann number M.

**Fig. 4:**
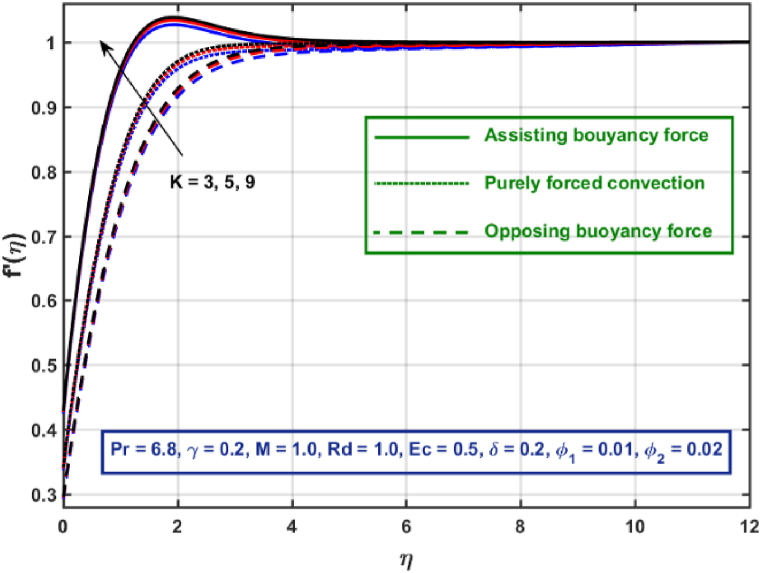
Vekocity profile for different values of K.

**Fig. 5:**
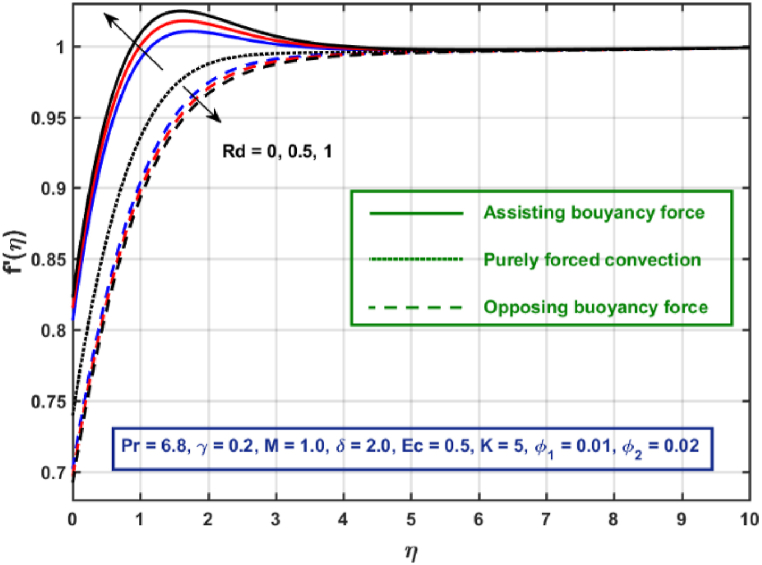
Vekocity profile for different values of Rd.

**Fig. 6:**
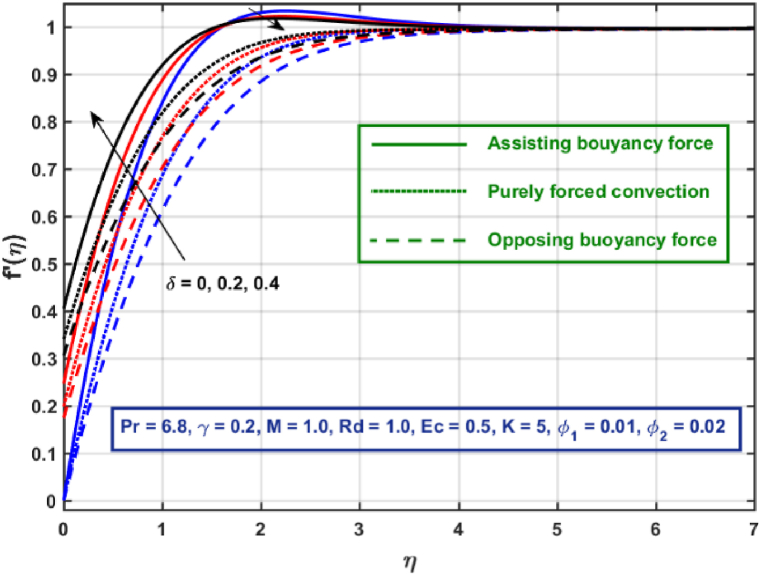
Velocity profile for different velocity slip parameter.

**Fig. 7:**
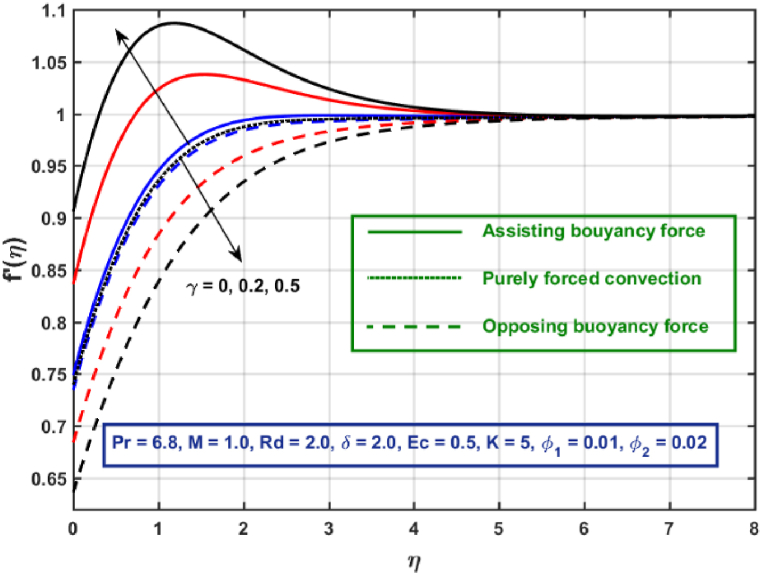
Velocity profile for different values of heat transfer coefficient.

**Fig. 8:**
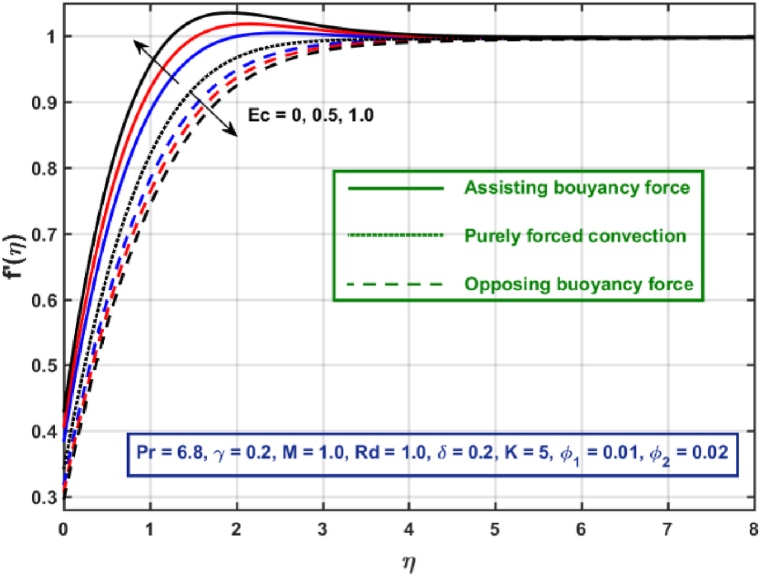
Velocity profile for different values of Eckert number .

**Fig. 9:**
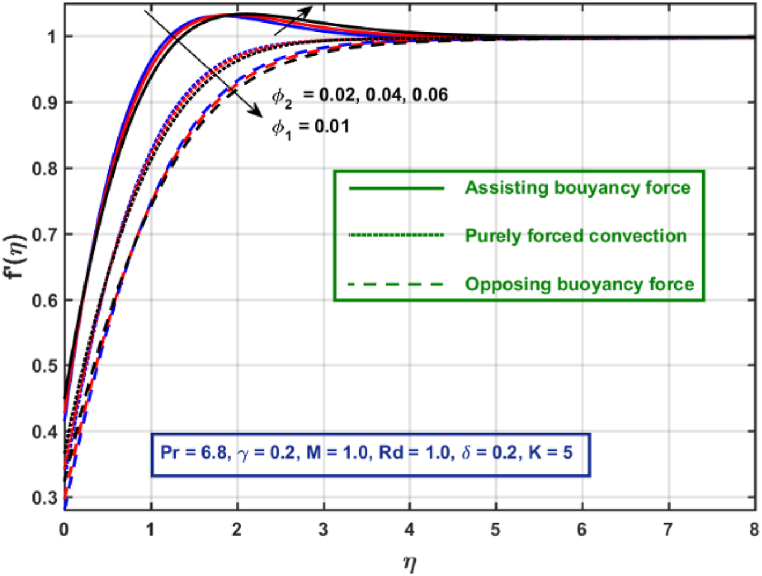
Velocity profile for different values of nanoparticles volume fraction.

**Fig. 10:**
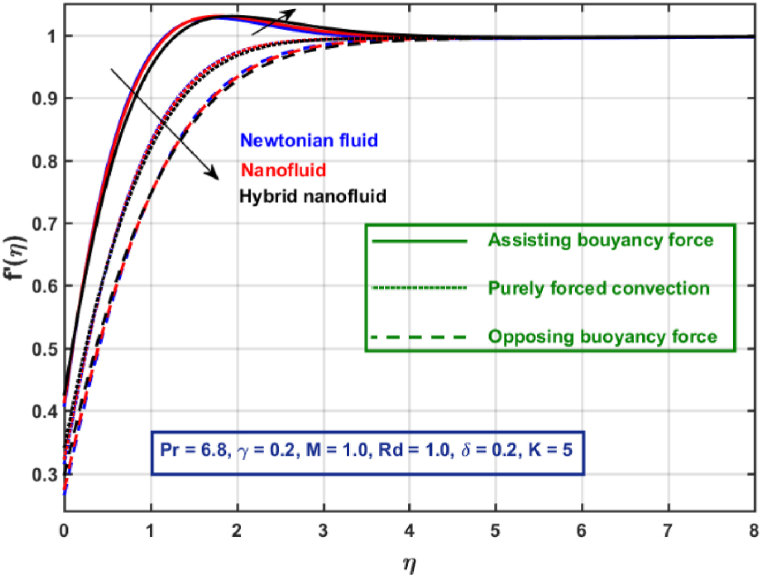
Velocity profile for different fluids.

Fig. 3, Fig. 4, Fig. 5, Fig. 6, Fig. 7, Fig. 8, Fig. 9, Fig. 10 are the graphical results for non-dimensional Velocity $f^{\prime }\left(\eta \right)$ plotted against non-dimensional distance η. From Fig. 3, it is depicted that hybrid CNTs fluid flow decelerates with an increase in Lorentz force magnitude. It is revealed that this trend remains the same in all the three scenarios i.e., in case of assisting, opposing buoyancy forces and purely forced convection, respectively. Moreover, the variation in velocity magnitude is significantly dominant when σ>0. This happens because Lorentz force always contribute as a resistive force which resist the fluid flow and subsequently fluid flow decelerates. The influence of medium porosity on fluid flow is shown in Fig. 4. It is observed that velocity upsurges for rising values of K in all the three scenarios. This happens mainly because the contribution of porosity parameter appears as a reciprocal in momentum equation and with the increase in K, a rise in fluid flow is witnessed. Physically K is the ratio of medium permeability to kinematic viscosity. The increasing estimates of K indicates a rise in medium permeability and a reduction in viscous forces which lead to accelerate fluid flow. Fig. 5 shows thermal radiation parameter impact on velocity. It is witnessed that velocity upsurges with an upsurge in Rd in presence of assisting buoyancy force and reverse behavior is observed in presence of opposing buoyancy force whereas no variation is recorded in presence of purely forced convection. Influence of velocity slip parameter δ on hybrid CNTs flow is displayed in Fig. 6. It is revealed that velocity slip parameter contributes to upsurge flow transport in all the three scenarios. However, in the case of favorable buoyancy force a decrease in velocity is reported with an increase in δ far away from the stretching surface. Fig. 7, Fig. 8 are plotted to examine the effect of heat transfer coefficient and Eckert number on velocity distribution. It is revealed that velocity upsurges with a rise in γ and Ec, when σ>0 an opposite trend is reported in case of opposing buoyancy force and purely forced convection. It is worth mentioning here that the upsurge in velocity is significantly prominent in case of assisting buoyancy force. The effect of Hybrid CNTs fluid volumetric fraction $\varphi_{2}$ on velocity is displayed in Fig. 9. It is observed that fluid transport decelerates with an increase in $\varphi_{2}$ for all the three scenarios. Moreover, this trend altered distant from the stretching surface presence of assisting buoyancy force. Fig. 10 is drawn for the comparison between Newtonian, CNTs nanofluid and Hybrid CNTs nanofluids. It is depicted that the decrease in velocity is prominent in presence of Hybrid CNTs nanofluid.

### 4.2 Temperature distribution

**Fig. 11:**
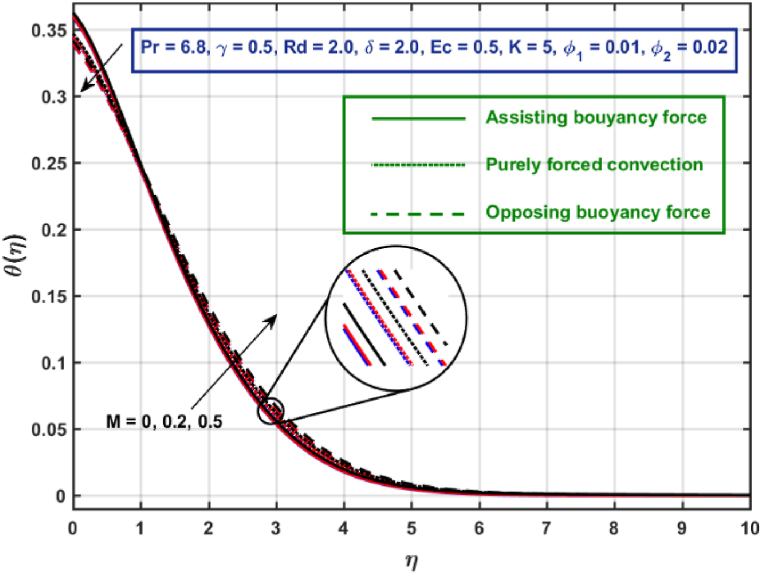
Temperature profile for different values of Hartmann number M

**Fig. 12:**
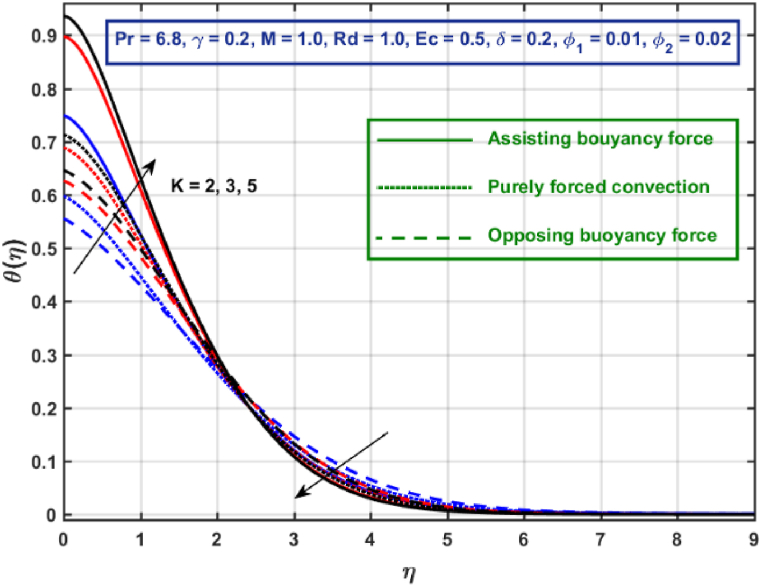
Temperature profile for different values of K.

**Fig. 13:**
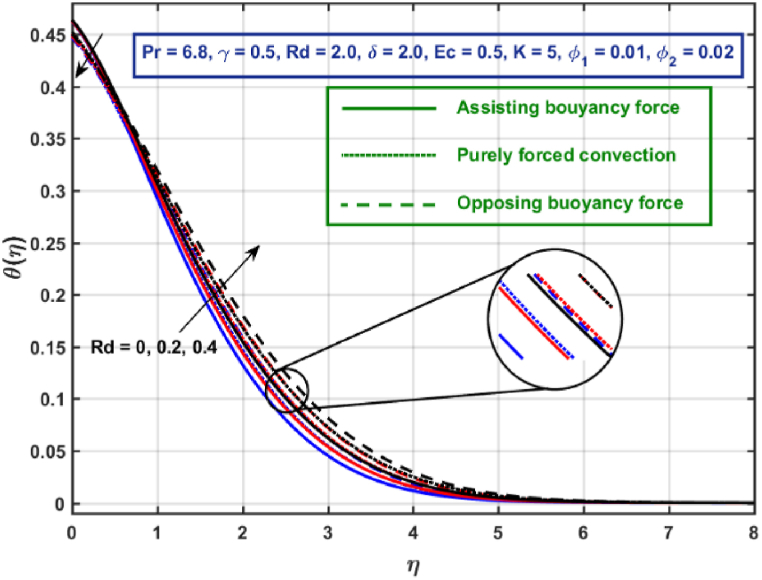
Temperature profile for different values of Rd.

**Fig. 14:**
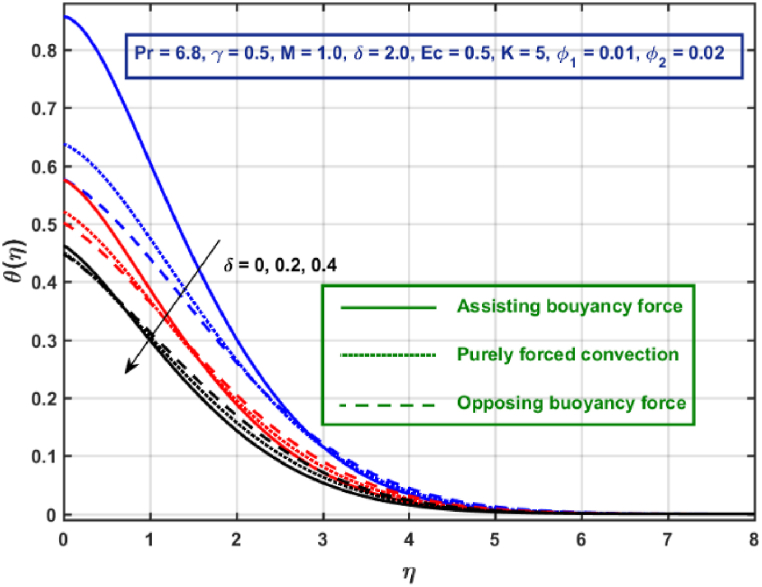
Temperature profile for different values of Slip parameter.

**Fig. 15:**
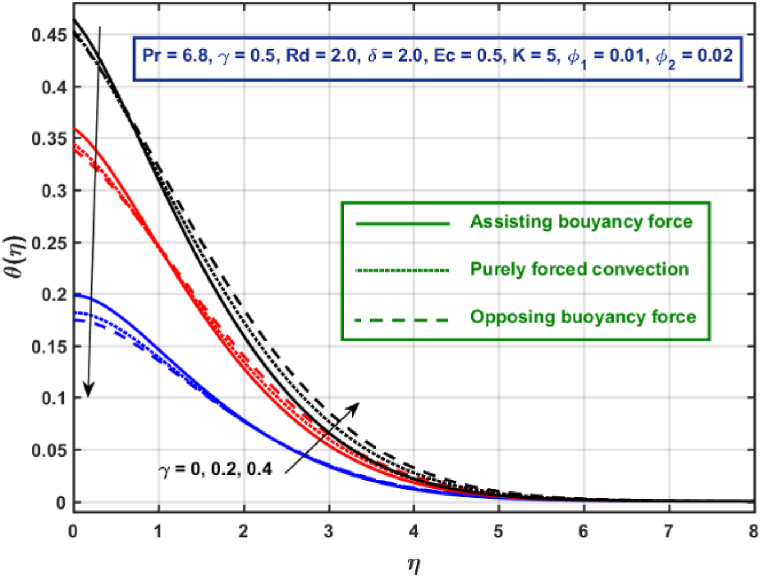
Temperature profile for different values of heat transfer coefficient.

**Fig. 16:**
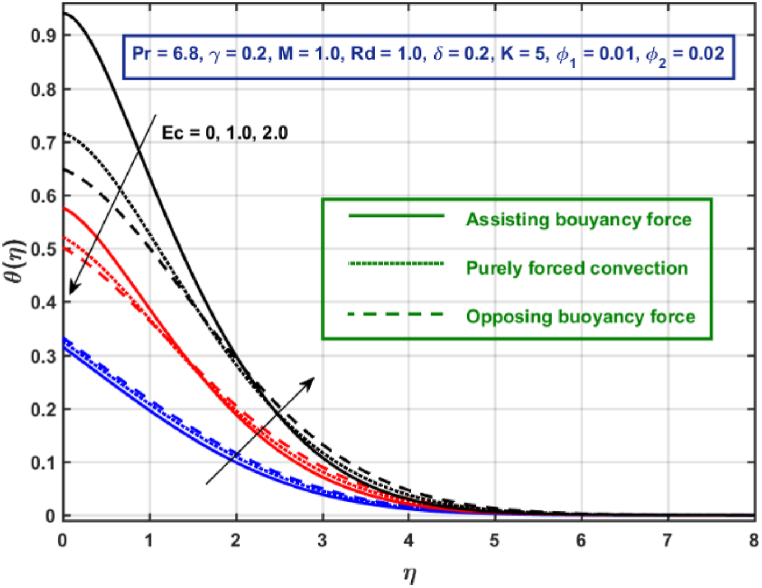
Temperature profile for different values of Ec

**Fig. 17:**
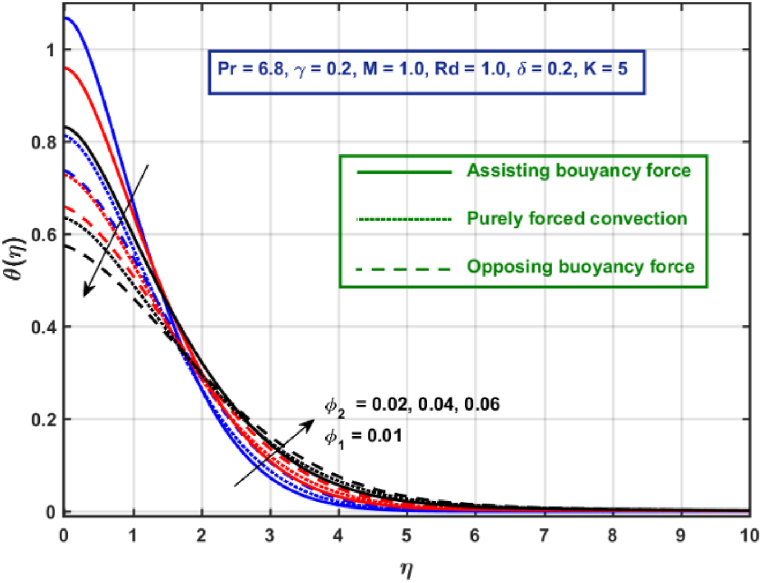
Temperature profile for different values of nanoparticles volume fraction

**Fig. 18:**
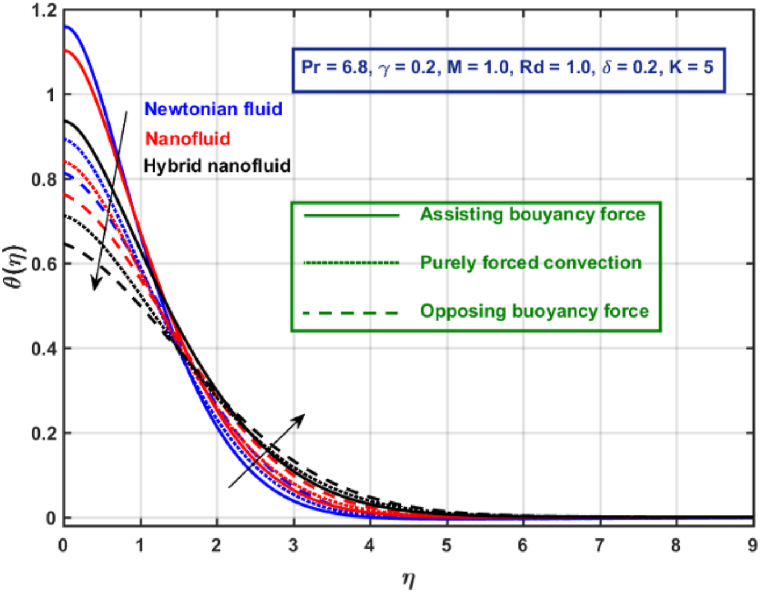
Temperature profile for different fkuids.

Fig. 11, Fig. 12, Fig. 13, Fig. 14, Fig. 15, Fig. 16, Fig. 17, Fig. 18 are the graphical results for non-dimensional velocity θ(η) plotted against non-dimensional distance η. From Fig. 11, it is evident that hybrid CNTs fluid temperature upsurges with an increase in external magnetic field magnitude. It is noted that this trend remains the same in all the three scenarios i.e., in case of assisting, opposing buoyancy forces and purely forced convection, respectively. Moreover, the maximum rise in temperature magnitude is recorded in the presence of opposing buoyancy force. The influence of medium porosity on temperature is displayed in Fig. 12. It is observed that temperature upsurges for rising values of K at stretching surface locality, however, this trend alters far away from the surface in all the three scenarios. Moreover, when σ>0, temperature variation is prominent in comparison to other scenarios. Fig. 13 is portrayed to explore the thermal radiation parameter effect on θ. It is seen that temperature upsurges for rising values of Rd in all the three cases. Influence of velocity slip parameter δ on hybrid CNTs temperature is displayed in Fig. 14. It is revealed that velocity slip parameter contributes to drop fluid temperature in all the three scenarios. However, in the case of opposing buoyancy force and purely forced convection an upsurge in temperature is reported with an increase in δ far away from the stretching surface. Fig. 15, Fig. 16 are plotted to examine the effect of heat transfer coefficient and Eckert number on temperature distribution. It is depicted that temperature drops at the stretching surface vicinity and this trend alters far away from the surface, for rising values of γ and Ec in all the three scenarios. This happens mainly because of the dominance of medium porosity and velocity slip effect near the surface which contribute to accelerate fluid flow and subsequently temperature drops at the stretching surface vicinity. The effect of Hybrid CNTs fluid volumetric fraction $\varphi_{2}$ on θ is displayed in Fig. 17. It is depicted that fluid temperature reduces for rising values of $\varphi_{2}$ near the surface and this trend alters far away from the surface, in all the three scenarios. Fig. 18 is drawn for the comparison between temperature distribution in the case of Newtonian, CNTs nanofluid and Hybrid CNTs nanofluids. It is depicted that the hybrid CNTs nanofluid existence influence heat transfer mechanism prominently.

### 4.3 Meaningful industrial parameters

**Fig. 19:**
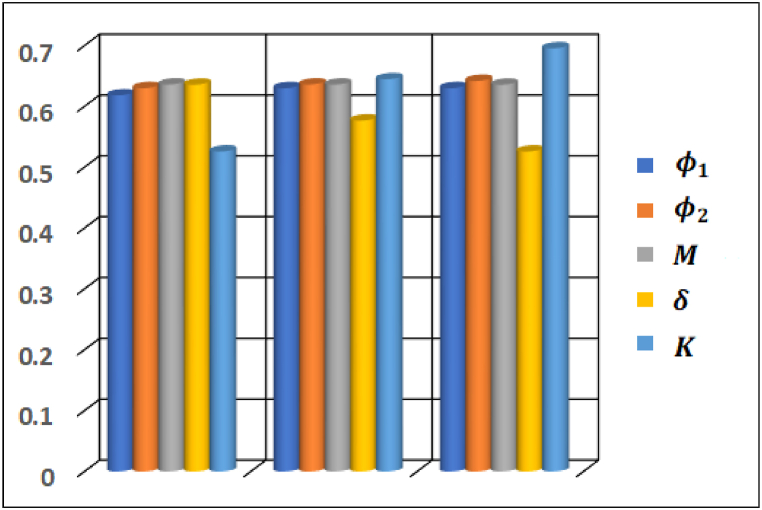
Variation in skin friction coefficient $C_{f}$.

**Fig. 20:**
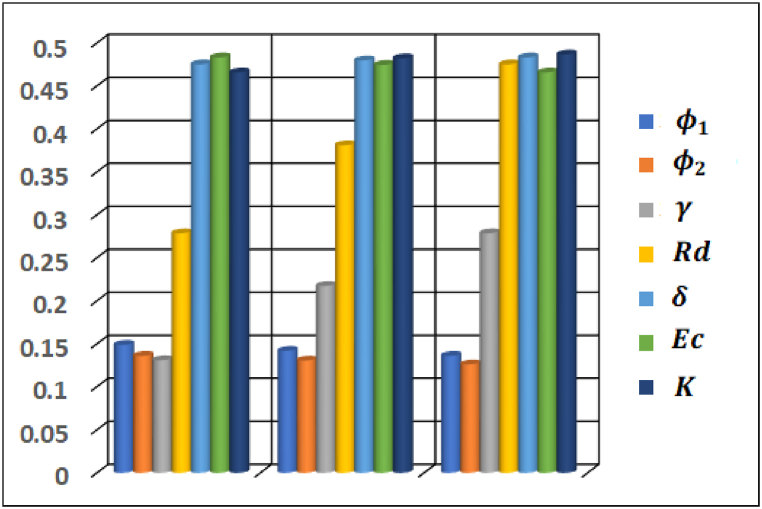
Variation in Nusselt number Nu.

The effect of prominent emerging parameters on important industrial quantities are studied and displayed through bar graphs plotted in Fig. 19, Fig. 20. It is depicted that volumetric fraction of CNTs $\varphi_{1}$ and $\varphi_{2}$ in hybrid nanofluid have opposite effect on $C_{f}$ and Nu contribute to upsurge surface drag force and lessen heat flux at the surface. Moreover, it is noted that magnitude of skin friction drops with an increase in Hartmann number and velocity slip parameter while medium porosity parameter influences $C_{f}$ in an opposite manner (see Fig. 19). Furthermore, Biot number, thermal radiation and medium porosity parameters contribute to enhance heat flux rate at the surface whereas Eckert number influences in an opposite manner.

Table 1 compares present results for local Nusselt number with those of Konishi et al. [3] for the case of no-porous wall s=0, in the absence of hybrid nanofluid i.e., $\varphi_{1}=0=\varphi_{2}$, buoyancy force i.e., σ=0, external magnetic field i.e., M=0, Eckert number Ec=0, and velocity slip δ=0. It is evident that these results are in excellent agreement.

**Table 1:** Comparison of present results for local Nusselt number in the limiting sense with those of Konishi et al. [3].

Pr	Present	Konishi et al. [3]
0.3	0.296874	0.296873
1.0	0.477558	0.477557
3.0	0.719279	0.719277
10.0	1.10672	1.10672

## 5 Conclusions

In the current study numerical investigation is perform to explore the impact of thermal stratification and medium porosity on gravity-coerced transport of hybrid CNTs down a vertical stretching sheet under the influence of an external magnetic field along with heat transfer analysis in presence of thermal radiation, viscous dispersal and joule heating effect. Moreover, velocity slip. The present computational study is performed for the distinct scenarios namely, (i) when σ>0 (physically this means presence of favorable buoyancy force), (ii) when σ=0 (physically this is case of purely forced convection) and (iii) when σ<0 (physically it represents the presence of opposing buoyancy force. The main key points include:

• External magnetic field and medium porosity influence the fluid flow as well as temperature distribution in an opposite manner.
• Presence of Hybrid CNTs nanofluid along with convective boundary and thermal radiation played a vital role to enhance fluid temperature.
• The presence of hybrid CNTs nanofluid and medium porosity contribute significantly in upsurging surface shear stress magnitude whereas, external magnetic field and velocity slip effects in an altered manner.
• Surface heat flux upsurges for rising values of thermal radiation, Biot number and medium porosity parameter whereas altered pattern is reported with an increase in volumetric fraction of hybrid CNTs nanofluid and Eckert number.

The present investigation may be extended for other types of nano and hybrid nanofluid in presence of nonlinear stretching and variable magnetic field keeping in view the processes involved in chemical engineering, nano and space technologies.

## Author contribution statement

Faisal Z. Duraihem; E.H Maraj; Noreen Sher; R Mehmood: Conceived and designed the analysis; Analyzed and interpreted the data; Contributed analysis tools or data; Wrote the paper.

## Data availability statement

No data was used for the research described in the article.

## Declaration of competing interest

The authors declare the following financial interests/personal relationships which may be considered as potential competing interests.

## Nomenclature

x,y(m): Cartesian coordinates
u,v(m/sec): Components of velocity profile
U(x)(m/sec): Free stream velocity
$g\;\left(m/\sec^{2}\right)$: Magnitude of gravitational force
$T_{f},T_{w},T_{0}\left(K\right)$: Fluid, wall, and ambient temperature
$\rho_{f},\rho_{hnf}\;\left(Kg/m^{3}\right)$: Fluid, hybrid nanofluid density
$\mu_{f},\mu_{hnf}\;\left(Kg/msec\right)$: Fluid, hybrid nanofluid viscosity
$\nu_{f},\nu_{hnf}\left(m^{2}/\sec\right)$: Fluid, hybrid nanofluid kinematic viscosity
$\beta_{f},\beta_{hnf}\left(K^{-1}\right)$: Fluid, hybrid nanofluid thermal expansion coefficient
${\tilde{\sigma }}_{f},{\tilde{\sigma }}_{hnf}\;\left(S/m\right)$: Fluid, hybrid nanofluid electrical conductivity
$k_{f},k_{hnf}\;\left(W/mK\right)$: Fluid, hybrid nanofluid thermal conductivity
${\left(C_{p}\right)}_{f},{\left(C_{p}\right)}_{hnf}\;\left(J/KgK\right)$: Fluid, hybrid nanofluid specific heat capacity
$\alpha_{f},\alpha_{hnf}\;\left(m^{2}/\sec\right)$: Fluid, hybrid nanofluid thermal diffusivity
$\varphi_{1},\varphi_{2}$: Nanofluid, hybrid nanofluid volumetric fraction
$B_{0}\;\left(Tesla\right)$: Magnitude of applied magnetic field
$k_{1}\;\left(Kg/m^{3}\right)$: Medium porosity
L(m): Ambient constant length
$h_{f}\;\left(W/m^{2}K\right)$: Convective heat transfer coefficient
$\sigma^{*}(W/m^{2}K^{4}$: Stefan Boltzmann constant
$k^{*}\left(m^{-1}\right)$: Rosseland mean absorption coefficient
$q_{r}\;\left(W/m^{2}\right)$: Radiative heat flux
η: Dimensionless space variable
$f^{\prime }$: Dimensionless fluid velocity
θ: Dimensionless fluid temperature
σ: Buoyancy force parameter
$Gr_{x}$: Local Grashof number
$Re_{x}$: Local Reynolds number
$M^{2}$: Magnetic parameter
Rd: Thermal radiation parameter
Pr: Prandtl number
Ec: Eckert number
δ: Velocity slip parameter
γ: Biot number
K: Medium porosity
$\tau_{w}\left(N/m^{2}\right)$: Shear stress at the surface
$q_{w}\;\left(W/m^{2}\right)$: Heat flux at the surface
$Cf_{x},Nu_{x}$: Local skin friction coefficient, Local Nusselt number
